# What else matters for endothelial dysfunction biomarkers in hemodialysis patients?

**DOI:** 10.1080/0886022X.2026.2644764

**Published:** 2026-03-23

**Authors:** Yu Zhao, Wenyun Wang, Zhanli Yang, Guirong Pu, Lijing Zhang, Chengji Zhao

**Affiliations:** a Department of Medicine, Northwest Minzu University, Lanzhou, PR China; b Department of the Second Hospital & Clinical Medical School, Lanzhou University, Lanzhou, PR China; c Department of Pediatric Surgery, Second Hospital of Lanzhou University, Lanzhou, PR China; d Department of Medicine Affairs, The Fourth people’s Hospital of Tianshui, Tianshui, PR China

Dear Editors,

We read with great interest the recent study by Lo et al. [1], which elegantly demonstrates that serum lipoprotein(a) (Lp(a)) levels are negatively correlated with the vascular reactivity index (VRI) and may serve as a potential biomarker for the early detection of endothelial dysfunction (ED) in maintenance hemodialysis (MHD) patients.This finding enriches the understanding of ED biomarkers in MHD patients. ED is highly prevalent in MHD patients, owing to the combined impact of uremic toxins, chronic inflammation, oxidative stress, and dialysis-related factors such as bioincompatibility. As Lp(a) exerts pro-inflammatory, pro-atherogenic, and pro-thrombotic effects by competing with plasminogen and activating monocytes [2], its role as a candidate biomarker for ED is biologically plausible and clinically relevant. However, ED is a complex multifactorial disorder triggered by a diverse array of pathways (Figure 1) [3], and single-biomarker assessment is insufficient to capture the full spectrum of endothelial injury in MHD patients. We aim to complement the findings of Lo et al. [1] by discussing additional ED-related biomarkers, therapeutic targets and key clinical confounders.

**Figure 1.**
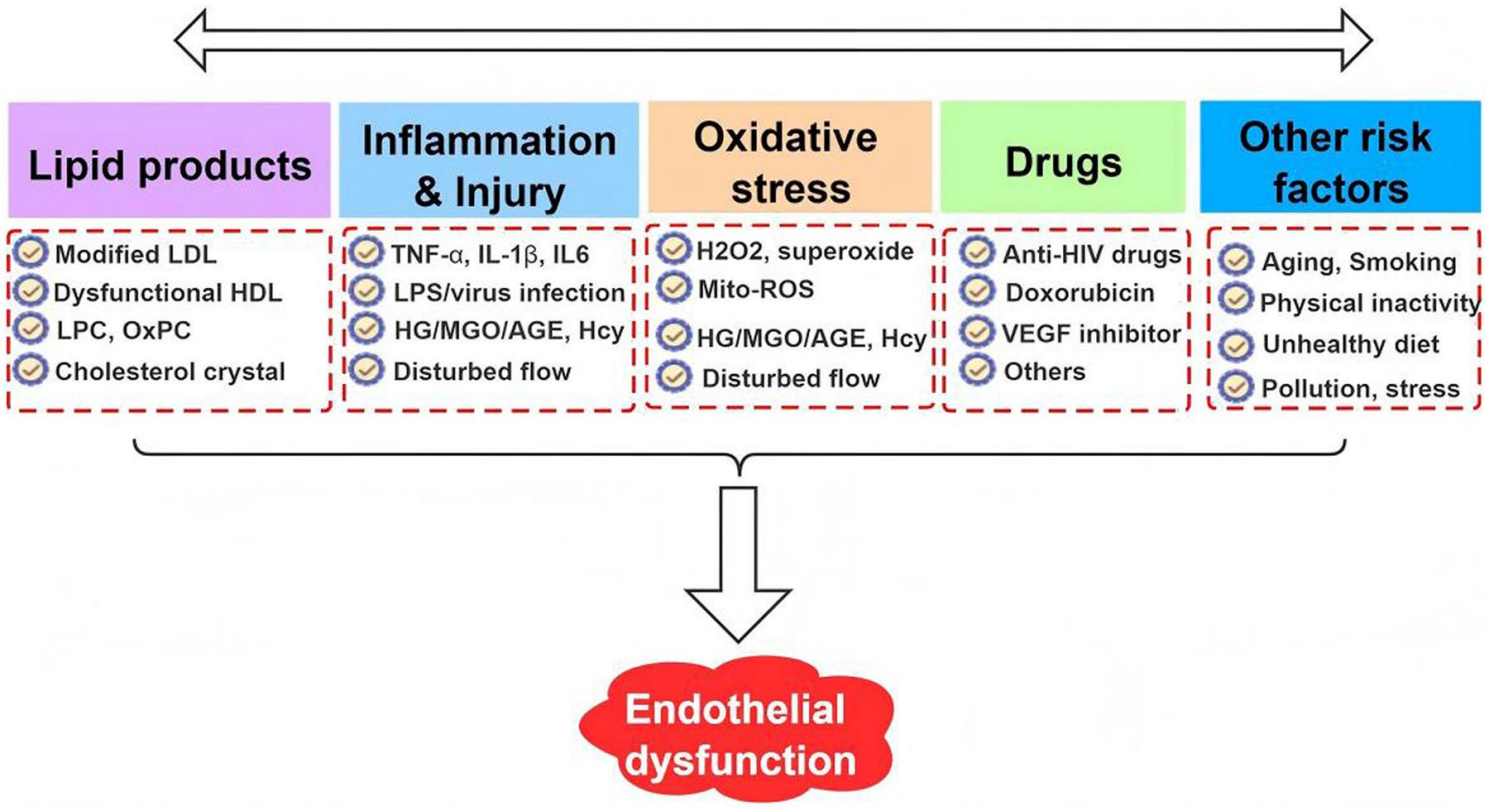
Triggers of endothelial dysfunction.There are different factors that can trigger endothelial dysfunction: (1) lipid [*via* modified LDL,dysfunctional high-density lipoprotein (HDL), lysophosphatidylcholine (LPC), oxidized phosphatidylcholine (OxPC), and cholesterol crystal]; (2) inflammation [by proinflammatory cytokines IL-1b, IL-6, TNF-a, and high-sensitivity c-reactive protein, LPS or virus infection, high glucose (HG)/methylglyoxal (MGO)/AGE, Hcy, and disturbed flow]; (3) oxidative stress including H_2_O_2_, superoxide, mitochondrial ROS, hyperglycemia-associatedstimuli, and disturbed flow; (4) different drugs such as anti–human immunodeficiency virus (HIV) drugs, anticancer agent doxorubicin, and vascularendothelial growth factor (VEGF) inhibitors may also trigger endothelial dysfunction; (5) other risk factors associated with endothelial dysfunctioninclude aging, smoking, physical inactivity, unhealthy diet, irradiation, air pollution (i.e. PM2.5), and psychologic stress etc. (from Ref. [3]).

First, monocyte chemoattractant protein 1 (MCP1), a key pro-inflammatory cytokine that mediates endothelial injury and is closely associated with ED in CKD patients, has been demonstrated to contribute to arteriovenous (AV) fistula failure in dialysis patients [4]. Myostatin, a member of the transforming growth factor-β superfamily secreted by mature muscle cells, exerts endotheliotoxic effects in the presence of uremic concentrations of indoxyl sulfate, amplifies the upregulation of MCP-1, and contributes to AV access complications [5]. Moreover, early AV fistula failure is closely related to ED, and different vascular access types (central venous catheter vs. AV fistula vs. AV graft) may have distinct impacts on baseline endothelial function due to differences in vascular injury and hemodynamic changes.

Second, endocan, a glycoprotein specifically secreted by activated endothelial cells, has emerged as a key mediator of inflammation, vascular smooth muscle cells proliferation, and angiogenesis, and serves as a novel biomarker for ED in chronic kidney disease(CKD) and MHD patients. Plasma endocan levels are significantly elevated in CKD patients (4.7 ng/mL, interquartile range(IR) 1.9–9.4 ng/ml) compared with controls (1.2 ng/ml, IR 1.1–1.5 ng/ml, *p* < 0.001), with values progressively higher across stages of CKD [6]. A recent study of 122 MHD patients further confirmed that the development of aortic stiffness was independently associated with endocan (OR 1.566, 95% CI 1.224 ∼ 2.002, *p* < 0.001), and logarithmically transformed endocan was an independent predictor of carotid-femoral pulse wave velocity (β = 0.405, adjusted R^2^ change = 0.152, *p* < 0.001) upon multivariate liner regression analysis [7]. However, endocan levels are not specific to ED, as they can also be influenced by systemic inflammation, oxidative stress and comorbid cardiovascular diseases.

Third, serum asymmetric dimethylarginine (ADMA), a well-recognized marker of ED and nitric oxide synthase inhibition, has been shown to respond to targeted pharmacological interventions in MHD patients. In a randomized, double-blind, placebo-controlled trial of 135 hypertensive MHD patients, 16-week treatment with ramipril (2.5 mg daily) resulted in a marked reduction in serum ADMA levels, indicating its potential to improve biomarkers of ED [8]. Similarly, a prospective, placebo-controlled, block-randomized, double-blinded study demonstrated that oral febuxostat significantly lowered serum ADMA levels and high-sensitivity C-reactive protein levels in MHD patients, with a direct ameliorating effect on oxidative stress [9]. Like endocan, ADMA levels are affected by multiple factors including systemic inflammation, liver and kidney function, and comorbidities. Notably, a randomized controlled trial of 50 MHD patients found that participants assigned 1:1 to receive niacin (500 mg/day) plus standard therapy for 3 months exhibited an 11.4% reduction in Lp(a) levels compared with the control group [10], providing preliminary evidence that targeting Lp(a) could be a viable approach for ED management.

Finally, building on the findings of Lo et al. [1] and the complementary perspectives presented in this commentary, future research on ED in MHD patients should aim to address the following key directions: (1) expand sample sizes and adopt multicenter study designs to enhance generalizability; (2) integrate multi-biomarkers (including Lp(a), MCP1, Myostatin, endocan, ADMA) and clinical confounders (e.g. vascular access type, dialysis vintage) to improve the accuracy of ED prediction; (3) conduct longitudinal studies to validate the causal relationship between biomarker levels (alone and in combination) and ED progression; and (4) explore the efficacy of combined therapeutic strategies in improving vascular outcomes in MHD patients.

In conclusion, Lp(a) is a valuable biomarker for ED in MHD patients. Nevertheless, single-biomarker strategies are inadequate to characterize the multifactorial pathogenesis of ED in this population. A multi-biomarker panel combining Lp(a), endocan, ADMA, MCP-1, and myostatin, together with clinical confounders such as vascular access, allows a more comprehensive and accurate assessment of ED than single biomarkers alone. This integrated strategy reflects multiple pathways of endothelial injury, including atherogenesis, endothelial activation, nitric oxide synthase inhibition, and inflammation, while accounting for clinical features specific to MHD patients, thus improving predictive value for ED. However, wide clinical application is currently limited by the high cost and lack of standardized assays for these biomarkers in routine dialysis settings. Future studies should focus on developing affordable, standardized detection methods to facilitate clinical translation.

## Data Availability

Data sharing is not applicable to this article as no new data were created or analyzed in this study.
